# Lung function and fractional exhaled nitric oxide among petroleum refinery workers

**DOI:** 10.1186/s12995-015-0080-7

**Published:** 2015-10-01

**Authors:** Sultan Ayoub Meo, Abdulrahman Hamad Alrashed, Abdulrahman Abdulaziz Almana, Yazeed Ibrahim Altheiban, Mohammed Saud Aldosari, Nawaf Faleh Almudarra, Sulaiman Ali Alwabel

**Affiliations:** Department of Physiology, College of Medicine, King Saud University, Riyadh, Kingdom of Saudi Arabia; Clinical Physiology, Department of Physiology (29), College of Medicine, King Khalid University Hospital, King Saud University, P.O. Box 2925, Riyadh, 11461 Saudi Arabia

**Keywords:** Crude oil, Lung functions, Fractional exhaled nitric oxide (FeNO)

## Abstract

**Background:**

Occupational and environmental exposure to petroleum refinery products poses a great threat to human health. This study aimed to assess the lung function and Fractional Exhaled Nitric Oxide (FeNO) among petroleum refinery workers.

**Methods:**

In this cross-sectional study, 112 participants (56 petroleum refinery workers with mean age 35.20 ± 1.62 years, and 56 age, weight, height, ethnicity and socioeconomically matched control subjects with mean age 30.02 ± 1.76 years) were recruited. A comprehensive clinical history and examination was conducted to decide whether to include in the study or not. Ventilatory lung function test parameters were recorded by using a Spirometer and Fractional Exhaled Nitric Oxide (FeNO) was measured by Niox Mino.

**Results:**

A significant decline in lung function parameters FEV1 (*p =* 0.0001), FEV1/FVC Ratio (*p =* 0.01), PEF (*p =* 0.0001), FEF 25 % (*p =* 0.0001), FEF-50 % (*p =* 0.012) was observed among oil refinery workers compared to their matched controls. However, there was no difference in the mean values of FeNO between the groups.

**Conclusion:**

Subjects working in the petroleum refinery have significantly impaired lung functions. However, there was no change in the values of FENO between the groups. The lung function impairment pattern provide evidence in the favor of an obstructive lung disease.

## Introduction

In Middle East, large number of petrochemical refineries are located mainly in the Gulf Cooperation Council (GCC) countries including Saudi Arabia, UAE, Qatar, Kuwait and Bahrain. These countries have added the most incremental petrochemical refinery capacity. People working in oil refineries are exposed to crude oil and wide variety of other petrochemical compounds. Crude oil is a complex combination of many chemical compounds; composed mainly of para-phenol, aromatic hydrocarbons [1], benzene, chromium, mercury, nickel, nitrogen, oxygen, iron, sulfur, toluene and xylene [2]. The fragrant hydrocarbons of toxicological attention are benzene, poly nuclear aromatic hydrocarbons [3] and traces of metals. Crude oil also contains large quantity of sulfur, light naphtha, heavy naphtha, and gasoline. The aromatic hydrocarbons are the main airborne exposure [1]. The petrochemical hydrocarbons compounds possess toxico-kinetc properties which become a cause of health risk [4, 5]. A large number of professionals work in the petrochemical industries. Oil refinery workers are prone to various pulmonary diseases secondary to inhalation of petrochemical compounds and lead to an immunological response mechanism causing hypersensitivity [4, 5]. Occupational and environmental exposure to petrochemical products pose threat to human health and have caused world-wide concerns.

Spirometry and Fractional Exhaled Nitric Oxide (FeNO) are mainly important in the workplace where occupational and environmental inhalation exposures can affect and cause respiratory diseases such as bronchial asthma and chronic obstructive pulmonary disease (COPD). Lung functions along with FENO are essential in the diagnosis of respiratory problems. Lung function and FENO measurements could be used for clinical settings especially for the early diagnosis of COPD development [6, 7]. Respiratory functions in addition to Fractional Exhaled Nitric Oxide (FeNO) in subjects working in petrochemical refineries have not been collectively and extensively studied. Therefore, this study aimed to determine the lung functions and Fractional Exhaled Nitric Oxide (FeNO) among petrochemical refinery workers.

## Subjects and methods

The present study was conducted in the Department of Physiology, College of Medicine, King Saud University, Riyadh, Saudi Arabia during the period Oct 2014 to April 2015. Ethical approval was obtained from the Department of Community Medicine, College of Medicine, KSU and informed consent was obtained from all the participants.

In this study, 112 participants (56 petroleum refinery workers and 56 control subjects) were selected. The refinery workers worked 8 h a day, five days in a week. The mean duration of exposure in the petrochemical refinery was 12.44 ± 1.49 years. All the subjects were adjusted for age, weight, height, ethnicity and socioeconomic and demographic status without history of any other occupational or industrial exposure known to cause respiratory illness. A comprehensive clinical history and examination of each of the subjects was conducted to decide whether to include in the research study or not. The control group was also selected in a similar way. The control group consisted of technicians working at the university, receptionists, secretaries and porters.

### Exclusion criteria

Subjects with identified cases of anemia, blood diseases, diabetes mellitus, chronic obstructive pulmonary diseases, bronchial asthma, malignancy and drug addicts were excluded from the study. Subjects who smoke cigarette or shisha were also excluded from the study [8, 9].

### Spirometry

The ventilatory lung function parameters were measured by using an electronic spirometer SPIROVIT SP-1 (Schiller, Switzerland). Lung function test parameters were recorded including Forced Vital Capacity (FVC), Forced Expiratory Volume in first second (FEV1), Forced Expiratory Ratio (FEV1/FVC %), Peak Expiratory Flow (PEF), Forced Expiratory Flow (FEF-25 %), Forced Expiratory Flow (FEF-50 %) and Forced Expiratory Flow (FEF-75 %). The established techniques in performing the various lung function tests for this study were based on the American Thoracic Society of Standardization 2005 [10]. After taking a comprehensive clinical history and anthropometric data, subjects were informed about the entire process. The subjects were encouraged to practice the procedure at their best possible efforts before doing the actual pulmonary function test. The participants performed the test in standing position without a nose clip and best values were recorded.

### Fractional exhaled Nitric Oxide [FeNO]

The Fractional Exhaled Nitric Oxide [FeNO] was determined by using a Niox Mino, Aerocrine, Solna, Sweden. The FeNO device was pre-calibrated for a programmed tests for 300 measurements. Tests were recorded at a fixed time of the day to minimize the diurnal variation [11]. The established procedures in performing FeNO test was based on the American Thoracic Society/ERS Standardization procedure for FENO [11]. Tests for the oil refinery worker were performed in the adjacent room in the oil industry and for control group all measurements were conducted in Physiology Department.

### Statistical analysis

The data were computed into the computer, analyzed by using the Statistical Package for Social Sciences [SPSS for Windows, version 21.0]. Unpaired student’s t-test (parametric test) was applied to test the difference in the means between the variables. The level of significance was considered at *p <* 0.05.

## Results

Table 1 presents the comparison of anthropometric and ventilatory lung function parameters between petroleum refinery workers compared to their matched control. Mean age of control subjects was 30.02 ± 1.76 (mean ± SEM) years, height 174 ± 0.93 cm (mean ± SEM) cm and weight 80.25 ± 2.68 (mean ± SEM) kg. On the other hand, for oil refinery workers, mean age was 35.20 ± 1.62 (mean ± SEM) years, height 170.75 ± 0.83 (mean ± SEM) cm and weight 78.36 ± 1.55 (mean ± SEM) kg. There was no difference in anthropometric parameters age, height and weight among the groups (Table 1). There was a significant decrease in lung function parameters among oil refinery workers including FEV1 (*p =* 0.0001), FEV1/FVC Ratio (*p =* 0.013), PEF (*p =* 0.0001), FEF 25 % (*p =* 0.0001), FEF-50 % (*p =* 0.012) compared to their age, height, weight, ethnicity and socioeconomic status matched control subjects (Table 1). No change in the mean value of FENO was found between the groups (Table 2).

**Table 1:** Comparison of anthropometric and lung function parameters between the oil refinery workers and control group

Parameter	Control (n=56)	Oil refinery workers (n=56)	p-value
Age (years)	30.02±1.76	35.20±1.62	0.320
Height (cm)	174±0.93	170.75±0.83	0.100
Weight (kg)	80.25±2.68	78.36±1.55	0.543
FVC (lit)	5.21±0.15	4.76±0.20	0.083
FEV1 (lit/sec)	3.99±0.14	3.08±0.16	0.0001
FEV1/FVC (%)	76.66±2.01	67.19±3.15	0.013
PEF (lit/ sec)	6.89±0.34	4.80±0.37	0.0001
FEF 25% (lit/sec)	6.39±0.33	4.45±0.36	0.0001
FEF 50% (lit/sec)	4.62±0.25	3.65±0.28	0.012
FEF 75% (lit/sec)	2.24±0.16	1.92±0.15	0.151

Note: Values are presented in Mean ± SEM

**Table 2:** Comparison of anthropometric and FeNO between the oil refinery workers and control group

Parameter	Control (n=56)	Oil refinery workers (n=56)	p-value
Age (years)	30.02±1.76	35.20±1.62	0.320
Height (cm)	174±0.93	170.75±0.83	0.100
Weight (kg)	80.25±2.68	78.36±1.55	0.543
FeNO (ppb)	28.14±2.53	22.98±1.74	0.096

Note: Values are presented in Mean ± SEM

## Discussion

Oil refineries produce a wide range of volatile agents and particulate matter into the environment. Subjects exposed to oil refinery substances have been associated with adverse outcomes and respiratory illnesses [12, 13]. In the current study, we found a significant decrease in lung function parameters including FEV1, PEF, FEV1/FVC %, FEF-25 % and FEF-50 % in petroleum refinery workers relative to their matched control group. Although, there was no change in the mean value of FENO between the groups.

In 2007, a Greek oil-tanker ran aground, resulting in a huge oil spill along the coastal areas of Karachi, and large number of people were involved in oil cleanup operation. Meo et al., [14] conducted a study on the subjects who were involved in the cleaning of oil at the costal belt side. Subjects involved in oil cleanup operation had significant decline in Forced Vital Capacity (FVC), Forced Expiratory Volume in first second (FEV1), Forced Expiratory Flow (FEF (25–75%)) and Maximum Voluntary Ventilation (MVV) compared to their matched controls. This impairment was reversible and lung functions parameters were improved when the subjects were withdrawn from the oil cleanup sites.

In another study, Meo et al., [15] reported a duration-response effect and shows that long term exposure to crude oil distinctly decreased the pulmonary function and subjects who were exposed to crude oil spill for more than 15 days showed a significant reduction in FVC, FEV1, F7EF25–75 % and MVV relative to their matched controls. In the present study, we found that subjects exposed to oil refinery had a significant decrease in lung function parameters including FEV1, PEF, FEV1/FVC %, FEF-25 % and FEF-50 % relative to their control group. Similarly, Jung et al., [16] reported that, adolescents who lived close to the oil spill area showed a decrease in FEV1. Our findings are in agreement to the previous studies conducted by Meo et al., [14], Meo et al., [15], and Jung et al., [16].

Kesavachandran et al. [17] reported that the pulmonary function parameters (FVC and FEV1) were decreased among petrol pump (or also named as gas stations) workers compared to predicted value. Similarly, Singhal et al., [18] assessed the respiratory ventilatory functions in petrol pump workers who were exposed to petrol vapors during the duty hours. The ventilatory functions including FVC and FEV1 were found to be decreased in the study group. Sandip et al., [19] found a significant reduction in FEV1, FVC, FEF 25–75 %, PEFR and PIFR in petrol pump workers. Furthermore, Madhuri et al., [20] measured the pulmonary function test in petrol pump workers (filling attendants) who were exposed to petrol vapors during duty hours. FVC, FEV1, FEV1/FVC %, FEF25, FEF75 % and PEFR were significantly decreased in petrol pump workers. Similarly, Sushil et al., [21] showed that the lung functions FVC, FEV1, MVV and PEFR were decreased in petrol pump workers as compared to controls. The studies conducted by Kesavachandran et al. [17], Singhal et al., [18], Sandip et al., [19], Madhuri et al., [20] and Sushil et al., [21] supported our point of view that subjects exposed to petroleum substances have impaired lung function parameters.

Minov et al., [22] conducted a cross sectional study in subjects employed in the coking unit of a petroleum refinery compared to an equal number of age, gender, and smoking habits matched office workers. The lung function tests results showed significantly lower MEF-50 and MEF-75 among petroleum refinery workers. Moitra et al., [23] reported that, subjects involved in manual cigarette lighter refilling with liquefied petroleum gas (LPG) have decrement in FEV1, FEV1/FVC Ratio. The adverse effect may be relevant to occupational group exposed to volatile hydrocarbons especially in employment sectors. Rusconi et al., [24] conducted a study on the effects of exposure to petroleum refinery emissions on lung function in children and adolescents living in a petrochemical polluted area. Children living in the reference zone showed an impaired lung function parameters FEV1 and FEF (25–75%). Petro-chemical substances are a complex mixture of aromatic hydrocarbons with high volatility. These substances reach the deeper parts of the lungs. Workers exposed to petro- chemical substances are at risk of petro-chemical vapour inhalation and they have more chances of chronic involvement of lungs as indicated by the results in the present study. The petro-chemical pollutants may alter the properties and concentration of surfactant and may thus contribute to the closure of small airways [25].

In the present study, we found a significant decrease in lung function parameters including FEV1, PEF, FEV1/FVC %, FEF-25 % and FEF-50 % in petroleum oil refinery workers compared to their matched control group. However, we did not find any difference in the mean value of FENO between the petroleum refinery workers and control group. While the reason for non significant FeNo is not known, it might be due to the difference in the subject selection. In the present study, we selected the petroleum refinery workers who were involved in the lab inside the refinery plant. These workers were exposed for a short time to crude oil as they were, most of the time, work inside the lab and were exposed to crude oil only while taking the samples or during their visit to the site. We believe that the level of exposure to petroleum compounds for the outdoor workers particularly who were involved in digging, cleaning and shifting the oil, would be much higher than that for indoor workers such as inside the lab. Hence we did not find any significant difference in FeNo values between the groups.

### Study limitations

The present study has few limitations that must be mentioned. One of the limitations of the present study is its cross-sectional nature that hampers to find any cause-and-effect relationships. We considered the subjects who were involved in the lab of the oil refinery, these subjects were not very appropriate subjects as most of the time they were inside the lab in the industry which reduces their actual exposure time in oil refinery. We calculate the power of the study, our study sample size is slightly low. Therefore we suggest that, in future large sample sized lung function along with FeNO measurement studies on the subjects with appropriate exposure to the petrochemical substances should be conducted to get the better inference.

## Conclusion

It is concluded that, subjects exposed to petrochemical refinery have significantly decreased in lung function parameters. However, our study could not detect any change in the mean value of FENO between the groups. The lung function pattern observed in this study provide an evidence in the favor of an obstructive lung disease. We believe, that, workplace exposure among petroleum refinery workers lead to lung function impairment. Kingdom of Saudi Arabia is one of the world largest oil producing countries, large numbers of people are involved in oil refinery. This study gives great insight into the health problems faced by oil refinery workers and there is a need to take more preventive measures to minimize the health hazards to the workers who are involved in the petroleum industries.
